# Beyond the dichotomy: understanding the overlap between atopic dermatitis and psoriasis

**DOI:** 10.3389/fimmu.2025.1541776

**Published:** 2025-02-10

**Authors:** Mengmeng Li, Jiangyi Wang, Qingfeng Liu, Youqing Liu, Wenyao Mi, Wei Li, Jingyi Li

**Affiliations:** ^1^ Department of Dermatology and Venerology, West China Hospital, Sichuan University, Chengdu, China; ^2^ Rare Diseases Center, West China Hospital, Sichuan University, Chengdu, China

**Keywords:** overlap, atopic dermatitis, psoriasis, psoriasis dermatitis, immunological networks

## Abstract

Atopic dermatitis and psoriasis have traditionally been considered distinct inflammatory skin diseases with unique pathogenic mechanisms. However, accumulating evidence suggests significant overlap in their immunological pathways, metabolic features, and microbiome characteristics, challenging this conventional dichotomy. This review comprehensively examines the complex relationship between psoriasis and atopic dermatitis, with particular emphasis on their shared and distinct pathogenic mechanisms. We analyze the immunological networks, metabolic pathways, and microbial factors contributing to their development and progression. The review expands upon the disease spectrum hypothesis and discusses the nomenclature for conditions exhibiting features of both diseases. We critically evaluate the clinical and histopathological characteristics of concomitant psoriasis and atopic dermatitis, highlighting recent advances in molecular diagnostics for accurate disease differentiation. Importantly, we propose standardized diagnostic criteria for psoriasis dermatitis and examine current therapeutic strategies for managing overlapping conditions. Recent developments in targeted therapies and their implications for treatment selection are thoroughly discussed. By synthesizing current evidence and identifying knowledge gaps, this review provides insights into the complex interplay between psoriasis and atopic dermatitis, aiming to guide clinical decision-making and future research directions in this evolving field.

## Introduction

Atopic dermatitis (AD) and psoriasis (PSO) represent two of the most prevalent chronic, relapsing, immune-mediated inflammatory skin disorders worldwide. Both conditions are characterized by recurrent flares and remissions that significantly impact patients’ quality of life. While traditionally considered distinct entities due to their different clinical presentations, underlying immune responses, and epidemiological features, recent evidence has challenged this perspective, revealing substantial overlap in their immunopathology and suggesting that their coexistence may be more common than previously recognized (1).

AD predominantly manifests in childhood, affecting 15-25% of children globally, with 7-10% of cases persisting into adulthood (2–5). The condition shows increased prevalence in industrialized countries and demonstrates strong associations with other atopic conditions, including asthma, allergic rhinitis, and food allergies (6). In contrast, PSO affects approximately 125 million individuals worldwide, with prevalence rates varying from 0.1% to 8% (7). While PSO typically emerges during adolescence or early adulthood, it can manifest at any age (8). In the past decades, AD and PSO were traditionally viewed as distinct and mutually exclusive entities, based on their apparently opposing immune pathways (9). Although AD is primarily driven by Th2-mediated responses, severe or treatment-resistant cases have shown involvement of interleukin-17 (IL-17), a cytokine typically associated with PSO pathogenesis (10). Similarly, elevated levels of interleukin-22 (IL-22), traditionally associated with the pathogenesis of PSO, have also been observed in AD, where they contribute to epidermal hyperplasia and barrier dysfunction (11, 12). A large-scale Korean cohort study following 3.9 million adults revealed that moderate-to-severe AD patients had the highest risk of developing PSO, suggesting a correlation between AD severity and PSO development (13). The therapeutic landscape has been transformed by targeted biologic therapies. For AD, biologics targeting the IL-4/IL-13 pathway, particularly dupilumab, have shown remarkable efficacy in moderate to severe cases (14). Similarly, PSO treatment has been revolutionized by IL-17 and IL-23 inhibitors, such as secukinumab and guselkumab (15). These therapeutic advances not only improve patient outcomes but also provide insights into the complex immunological mechanisms underlying both conditions.

A literature search was conducted on the PubMed database using a range of search terms, including “atopic dermatitis,” “psoriasis,” “coexistence of eczema and psoriasis,” “concomitant psoriasis and atopic dermatitis,” “eczema in psoriatico (EIP),” “psoriasis dermatitis (PD),” “overlapping features of psoriasis and atopic dermatitis,” “eczematous psoriasis/eczematized psoriasis,” “psoriasiform paradoxical reactions (P-PRs),” “eczematous paradoxical reactions (E-PRs),” “dupilumab-associated psoriasis,” and “psoriasiform manifestations (DAPs/PsM),” “Pathogenic Mechanisms,” and “Treatment.” Only papers published in the English language before May 2024 were considered. This review aims to elucidate the emerging concept of overlap between AD and PSO, with a focus on their shared characteristics. It emphasizes recent advances in understanding their immunopathology, the mechanisms enabling their coexistence, and the implications for therapeutic strategies.

## Shared pathogenic mechanisms of atopic dermatitis and psoriasis

### Divergent immune mechanisms: beyond the Th1/Th17 versus Th2 paradigm

**Figure 1:**
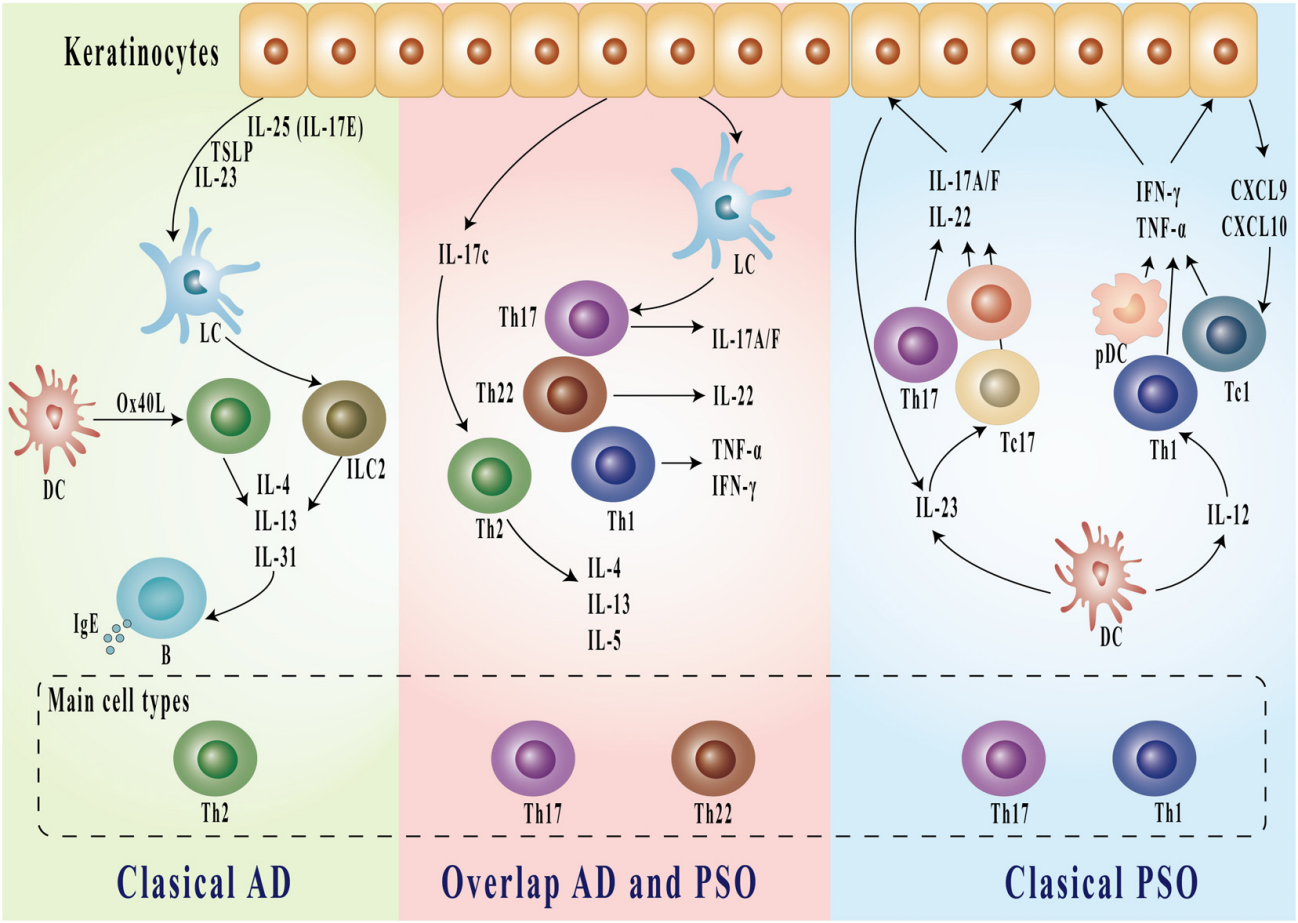
Immunologic pathways in classical AD, overlapping AD and PSO, and classical PSO. Classical AD is characterized by Th2-dominant inflammation, where IL-4, IL-13, and IL-31 from Th2 cells stimulate B cells to produce IgE. In the overlapping AD and PSO, both Th17 and Th22 cells are involved, producing IL-17A/F, IL-22, TNF-α, and IFN-γ. Classical PSO features predominant Th17 and Th1 responses, with production of inflammatory cytokines including IL-17A/F, IL-22, TNF-α, and IFN-γ, as well as chemokines CXCL9 and CXCL10. Keratinocytes interact with these pathways by releasing various cytokines and mediators including TSLP, IL-25, and IL-23. AD, atopic dermatitis; PSO, psoriasis; DC, dendritic cells; LC, Langerhans cells; pDC, plasmacytoid dendritic cells; Th, T helper cells; IL, interleukin; IFN, interferon; TNF, tumor necrosis factor; TSLP, thymic stromal lymphopoietin; CXCL, C-X-C motif chemokine ligand.

The immunopathogenesis of these conditions exhibits both distinct and overlapping characteristics. The traditional view of AD and PSO has emphasized their distinct immunological profiles. AD is primarily driven by Th2-mediated immune responses, with cytokines such as IL-4, IL-13, and IL-31 playing pivotal roles in disrupting skin barrier function, promoting IgE production, and modulating inflammatory pathways (16–20). The resulting chronic inflammation and impaired skin barrier function contribute to secondary infections and exert a substantial psychosocial impact on affected individuals (21). While the pathogenesis of AD is not completely understood, it is influenced by a complex interplay of genetic susceptibility, environmental factors, and psychological stress (5). PSO is primarily characterized by dysregulation of the IL-17/23 axis and a disordered T helper (Th)1/Th17 cell-mediated immune response, in stark contrast to the inflammatory profile of AD, which is driven by dysregulation of the IL-4/IL-13 axis and a Th2/Th17/Th22 cell-mediated response. Recent studies highlight that AD is an IL-13-dominant condition, exhibiting greater molecular heterogeneity compared to psoriasis (19). In psoriasis, the predominant Th1 and Th17 pathways are associated with elevated levels of IFN-γ, TNF-α, IL-17, and IL-22, which promote keratinocyte hyperproliferation and the formation of characteristic psoriatic plaques (18, 22, 23). These cytokines promote keratinocyte hyperproliferation and contribute to systemic inflammation, underscoring their central role in the disease’s inflammatory cascade (24). A critical pathogenic triad exists among dendritic cells, Th17 cells, and keratinocytes. Dendritic cell-derived TNF-α and IL-23 promote the differentiation of Th17 cells, which subsequently produce inflammatory cytokines that fuel disease progression (25). However, recent advances in immunological research have unveiled a more intricate landscape. The finding that IL-17, a cytokine traditionally linked to PSO pathogenesis, also plays a pivotal role in certain subsets of AD—particularly in severe or treatment-resistant cases—challenges the conventional paradigm (11, 26). A recent study identified nummular eczema (NE), a chronic inflammatory skin condition characterized by pruritic, discoid-shaped lesions, as exhibiting a codominant Th2/Th17 immune response, supporting its classification as a subtype of AD (27). Conversely, the identification of IL-4 and IL-13 involvement in a subset of PSO patients exhibiting atopic features suggests a more nuanced understanding of these conditions (28). Liu et al. elucidate distinct cytokine profiles in psoriasis, notably elevated Th17-associated cytokines such as IL-17F and IL-26, in contrast to the Th2-dominant expression seen in AD. Markers like CXCL13 show strong correlations with disease severity and exhibit considerable variability across patients, underscoring the molecular heterogeneity within psoriasis. These findings advocate for therapeutic strategies tailored to individual immune profiles, challenging the conventional Th1/Th17 versus Th2 framework ​ (29). A study examining immune cell profiles in psoriasis and atopic dermatitis reveals that while chronic cases typically exhibit a Th1 dominance in psoriasis and Th2 dominance in AD, these distinctions become less pronounced during erythrodermic exacerbations. The lack of significant differences in Th1, Th2, Th17, and Th22 cells in erythrodermic cases suggests shared inflammatory mechanisms beyond the traditional Th1/Th2 paradigm, supporting a more nuanced understanding of their immunological landscape and potential convergent pathways. This insight aligns with current perspectives on overlapping pathogenic mechanisms between psoriasis and atopic dermatitis in acute phases of disease (30). These findings suggest that immune responses in AD and PSO exist along a spectrum rather than as strictly dichotomous entities, with a complex interplay between Th1, Th2, and Th17 pathways. Notably, a distinct Th17/IL-23 signature has been identified in specific AD cases, particularly among pediatric patients and those of Asian descent (11, 31–33). These observations emphasize the need for a more sophisticated understanding of the immune pathways involved, fundamentally challenging the traditional dichotomy between Th1/Th17 and Th2 responses. The main immunopathogenesis of AD and PSO is shown in **Figure 1**.

### Skin barrier dysfunction: a shared pathophysiological feature

Skin barrier dysfunction represents a fundamental pathophysiological feature common to both AD and PSO, although the underlying mechanisms driving this dysfunction differ significantly between the two conditions. Understanding these distinct yet interconnected pathways provide crucial insights into disease pathogenesis and potential therapeutic approaches.

Both conditions exhibit significant skin barrier dysfunction, albeit through different mechanisms (5, 9). In AD, barrier compromise is primarily attributed to genetic mutations, particularly in the *filaggrin (FLG)* gene, leading to increased transepidermal water loss (TEWL) and heightened susceptibility to environmental allergens and microbial colonization, notably by Staphylococcus aureus (34–36). These defects create a self-perpetuating cycle in which *FLG* deficiency not only disrupts the physical barrier but also intensifies Th2-driven inflammation, further impairing barrier integrity (37). In contrast, PSO-related barrier dysfunction stems from dysregulated keratinocyte activity, largely mediated by inflammatory cytokines such as IL-17 and IL-22, leading to the formation of characteristic psoriatic plaques (38, 39). Although *FLG* mutations are not central to PSO pathogenesis, altered expression of other barrier-related proteins, including loricrin and involucrin, has been observed within psoriatic lesions, suggesting that barrier compromise in PSO may be mediated by different molecular pathways (40, 41).

This shared characteristic of barrier dysfunction underscores a potential overlap in the inflammatory pathways of AD and PSO, contributing to chronic inflammation and supporting a growing body of evidence for immunological interplay between these conditions. The convergence of these mechanisms highlights the evolving understanding of their pathogenesis, with implications for therapeutic approaches that may target these shared pathways.

### Shared genetic susceptibility and comorbidities

Genetic predisposition plays a fundamental role in the pathogenesis of both AD and PSO, with each condition exhibiting distinct yet potentially overlapping genetic signatures. In AD, mutations in the *FLG* gene are significant risk factors, particularly among European populations (42). *Loricrin (LOR)* and *involucrin (IVL)* also play significant roles in the pathogenesis of AD. These findings have been consistently supported by genome-wide association studies (GWAS) and meta-analyses (43). In contrast, PSO demonstrates strong genetic associations with *HLA-Cw6* and immune-related genes such as *IL23R* and *IL12B*, which are essential in regulating Th17 and Th1 pathways (44). Notably, recent GWAS have identified shared genetic loci between AD and PSO, suggesting common underlying pathogenic mechanisms (45).

The systemic nature of AD and PSO is further evidenced by their distinct patterns of comorbidities. AD frequently coexists with atopic conditions like asthma, allergic rhinitis, and food allergies (46), while PSO is commonly associated with metabolic syndrome, obesity, and inflammatory diseases mediated by cytokines, such as cardiovascular conditions (47–49). The comorbidities and their corresponding inflammatory mediators are summarized in **Table 1**.

**Table 1:** The comorbidities of PSO and their corresponding inflammatory mediators.

Key comorbidities	Inflammatory cytokines
Nonalcoholic fatty liver disease	TNF-α
Cardiovascular diseases	TNF-α, IL-17, and IL-6
Diabetes mellitus	TNF-α, IL-6, IL-1β, IL-17, and IL-18
Psoriatic arthritis	TNF-α, IL-1, IL-17, IL-22, and IL-23
Inflammatory bowel disease	TNF-α, IFN-γ, IL-17, and IL-23
Obesity	TNF-α, IL-17A, and IL-6

In AD, atopic comorbidities, including food allergies, asthma, and rhinitis, often arise early, whereas non-atopic conditions typically appear later in disease progression (50). Although cardiovascular diseases are classified as non-atopic comorbidities in AD, this classification remains under debate (51–55). Additionally, AD is linked with conditions like alopecia areata (56), vitiligo (57), and neuropsychiatric disorders (58, 59), although the mechanistic basis of these associations remains unclear. Both AD and PSO are associated with elevated risks of depression and anxiety, likely due to the chronic visibility of skin lesions and the subsequent impact on quality of life (60).

The similarities in comorbid profiles and shared genetic loci underscore a complex interplay between the pathogenesis of AD and PSO, which may extend to common immunological pathways and comorbid risks, thereby supporting a more integrated approach to understanding these conditions.

### Similarities and differences in microbiome and metabolism

Recent advances in microbiome research have unveiled intricate relationships between gut microbiota alterations and inflammatory skin conditions, particularly AD and PSO. Chen et al. (61) provide a comprehensive review of these microbiome alterations, noting both shared features and distinct microbial profiles across AD and PSO.

Numerous studies have shown that the skin microbiome plays a significant role in the onset and progression of AD and PSO (61). However, notable differences exist in their bacterial alterations. *Streptococcus* is the dominant microbe in psoriatic lesions, strongly linked to guttate and chronic plaque psoriasis, but is typically reduced in AD (62, 63). Anaerobic bacteria such as *Lactobacillus* spp. and *Finegoldia* spp. are decreased in AD due to epidermal barrier dysfunction, whereas *Finegoldia* spp. increases in psoriatic lesions (64). *S. aureus* colonization is common in both diseases and correlates with severity (65). In AD, *S. aureus* superantigens (e.g., TSST-1, SEA, and SEB) induce IgE production and mast cell degranulation, aggravating inflammation (62). In psoriasis, α-type phenol-soluble modulins (PSMs) drive Th17-mediated inflammation, contributing to epidermal thickening and neutrophil infiltration. Additionally, *S. aureus* sheds lipopolysaccharides and lipoteichoic acid, maintaining psoriasis-associated chronic inflammation (66, 67).

Both AD and PSO demonstrate significant associations with gut dysbiosis, although the specific microbial signatures differ markedly. In AD, dysbiosis manifests as an increase in potentially pathogenic genera, particularly *Klebsiella* and *Escherichia coli*, accompanied by a reduction in beneficial bacteria such as *Bacteroides* and *Clostridium* (68–70). Interestingly, patients with transient AD exhibit a distinct profile characterized by elevated levels of *Akkermansia* and *Bifidobacterium* (68–70). These alterations in microbial communities may contribute to the characteristic dysregulated immune responses and chronic inflammation observed in AD. Meanwhile, PSO is associated with increased levels of *Faecalibacterium*, *Bifidobacterium*, and several genera within the *Actinobacteria* and *Firmicutes phyla*, alongside reduced levels of *Lachnospiraceae* and *Bacteroides* (71–73). *Faecalibacterium prausnitzii* has emerged as a particularly relevant marker for PSO-associated dysbiosis. Despite these distinct microbial compositions, both conditions display a notable consistency in microbiome structure, with neither demonstrating significant changes in alpha diversity—indicating stable overall richness and evenness of microbial communities. However, beta diversity analyses reveal disease-specific clustering patterns, distinguishing AD and PSO patients from healthy controls (70, 74). Recent research has demonstrated significant depletion of *Eubacterium rectale* in both PSO and psoriatic arthritis (PsA) patients, with notably lower abundances observed in PsA compared to PSO patients. Furthermore, two Alistipes species showed marked reduction in psoriatic patients. These microorganisms are pivotal in carbohydrate metabolism pathways, primarily generating short-chain fatty acids that exert anti-inflammatory effects (75).

In addition to microbial composition, metabolomic studies have identified various altered metabolites in both AD and PSO, although their precise roles in disease pathogenesis are not yet fully understood. The presence of gut and skin dysbiosis in both conditions highlights potential microbiome contributions to disease comorbidities and complications; however, the exact impact remains elusive due to confounding factors in study designs. Future research should prioritize controlling these variables to clarify the relationship between microbiome imbalances and the inflammatory pathways implicated in AD and PSO pathogenesis (61).

## Clinical presentation and histopathology of AD and PSO

**Figure 2:**
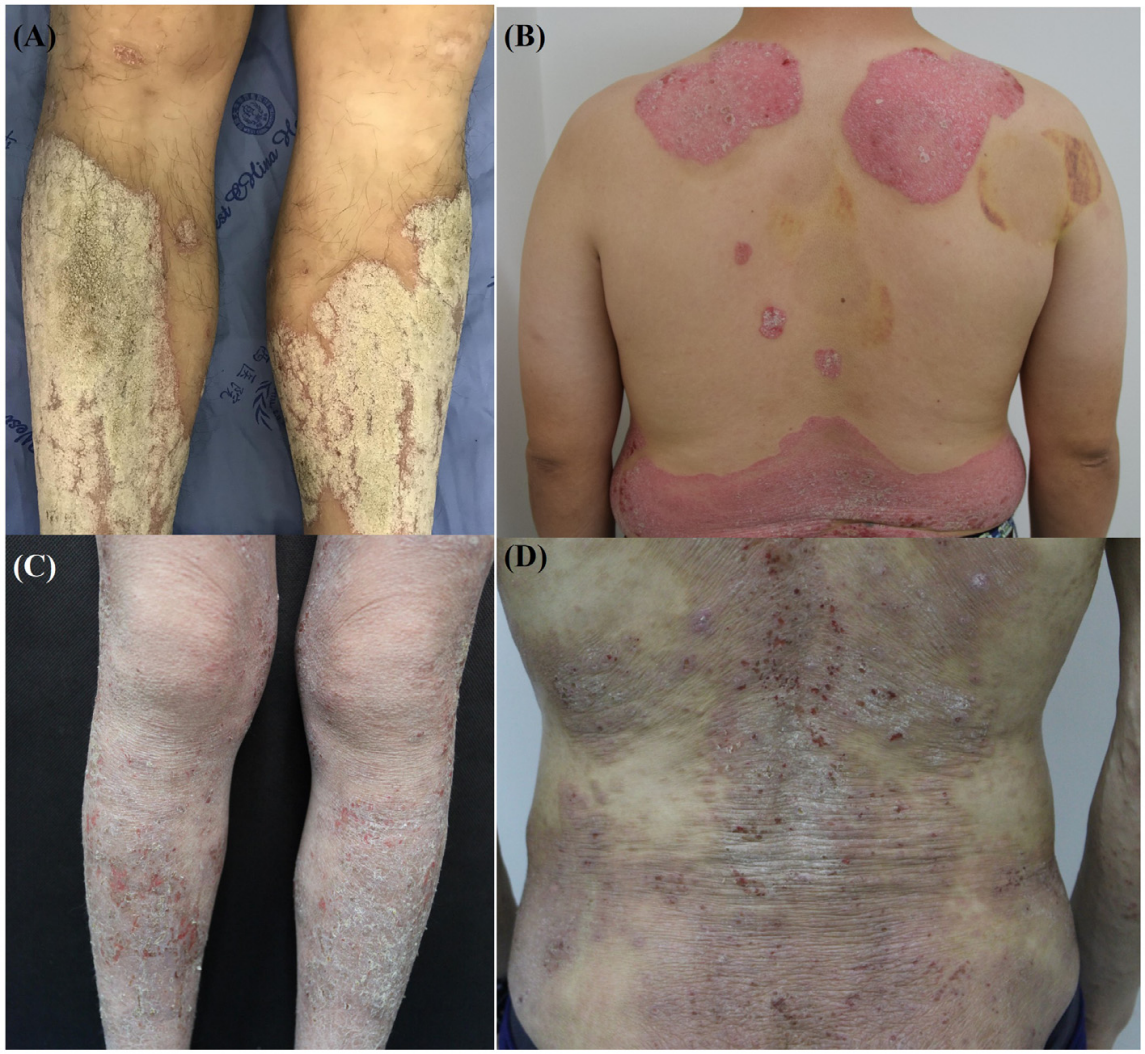
Clinical manifestations of atopic dermatitis and psoriasis. **(A, B)** Well-demarcated erythematous plaques with silvery scales on the lower extremities and back, characteristic of psoriasis. **(C, D)** Xerotic skin with erythematous patches, excoriations, and scaling on the lower extremities and back, typical features of atopic dermatitis.

AD and PSO present with distinctive clinical morphologies and distribution patterns that are essential for differential diagnosis. Clinically, AD primarily appears as intensely pruritic, erythematous patches and plaques, often accompanied by xerosis, excoriations, and lichenification due to chronic itching and scratching cycles (76). AD lesions commonly affect flexural areas, such as the antecubital and popliteal fossae, and frequently involve the face, neck, and dorsal aspects of the hands in adults (2). In contrast, PSO is characterized by well-demarcated, erythematous plaques covered with silvery-white scales, with pruritus typically less intense than in AD (77). These psoriatic lesions often manifest on extensor surfaces, including the elbows, knees, and scalp, although they may appear on any anatomical site (78). Clinical manifestations with AD and PSO are shown in **Figure 2**.

Histopathologically, AD and PSO exhibit distinct features crucial for differential diagnosis. In AD, histology displays marked spongiosis (intercellular edema within the epidermis) and hyperkeratosis, with the granular layer typically preserved or thickened. Chronic AD lesions often exhibit lichenification due to repeated scratching, leading to significant epidermal thickening. The dermis shows perivascular lymphocytic infiltration, with fibrosis occurring in longstanding cases (9). In contrast, PSO reveals prominent epidermal hyperplasia, elongation of rete ridges, parakeratosis (the presence of nuclei within the stratum corneum), and loss of the granular layer. A hallmark of PSO is Munro’s microabscess-clusters of neutrophils within the stratum corneum-along with dilated capillaries in the papillary dermis, contributing to the erythematous appearance of psoriatic plaques (18).

In summary, these histopathological differences-such as spongiosis and a preserved granular layer in AD versus parakeratosis and neutrophil presence in PSO -underscore the diagnostic distinctions between the two conditions, offering critical insights into their underlying pathologies.

### Clinical subtypes and coexistence patterns of AD and PSO

AD presents as erythematous, excoriated lesions accompanied by intense pruritus, whereas PSO is typically characterized by well-demarcated plaques covered with silvery scales. Despite these distinct clinical presentations, both conditions share common pathophysiological features, including immune cell infiltration, cytokine-driven inflammation, and compromised skin barrier functions (79, 80). AD and PSO also encompass various subtypes; AD is classified into multiple subtypes based on factors such as IgE levels, ethnicity, age, and clinical characteristics (**Table 2**) (81), while PSO most commonly manifests as chronic plaque psoriasis, the predominant form (77). The presence of erythrodermic features in AD can complicate diagnosis, as these may be difficult to distinguish from erythrodermic psoriasis. Research has underscored an immunological overlap between the two conditions, particularly involving helper T-cell subtypes Th17 and Th22 (29). This immunological interplay suggests that AD and PSO, along with their subtypes, may exist along a broader disease spectrum, with overlapping features.

**Table 2:** Subtypes of PSO and AD.

	Classification terminology	Principal classification approach	T Cell/Cytokine Skewing
Subtypes of atopic dermatitis	Extrinsic and Intrinsic subtypes	IgE levels and IgE specific to protein Antigens	Th2 *vs*. Additional Th1/T22/Th17
	European American and Asian subtypes	Ethnicity	Th2 *vs*. Additional Th17/IL-19
	Pediatric, adult and Elderly subtypes	Age bracket	Th2 *vs*. Th1/CLA− Th2/Th17/Th22
	Phenotype and Endotype	Clinical Manifestations and Molecular mechanisms	
Subtypes of psoriasis	chronic plaque or psoriasis vulgaris, guttate, erythrodermic, and pustular psoriasis		
	inverse or flexural psoriasis, palmoplantar psoriasis, sebopsoriasis, and nail psoriasis.		

CLA, Cutaneous Lymphocyte Antigen.

The coexistence of AD and PSO was first documented in 1992 (82), but initially, it received limited academic attention. Subsequent reports in the literature (82–87) (**Table 3**) have described cases of concurrent AD and PSO. However, the terminology used to describe their coexistence remains inconsistent and often ambiguous. Terms such as “coexistence of eczema and psoriasis,” “concomitant psoriasis and atopic dermatitis,” “eczema in psoriatico (EIP),” “psoriasis dermatitis (PD),” “overlapping features of psoriasis and atopic dermatitis,” “eczematous psoriasis/eczematized psoriasis,” “psoriasiform paradoxical reactions (P-PRs),” “eczematous paradoxical reactions (E-PRs),” “dupilumab-associated psoriasis,” and “psoriasiform manifestations (DAPs/PsM)” have all been used. This inconsistency highlights the need for a more standardized and precise nomenclature to describe the overlapping and coexisting presentations of these conditions.

**Table 3:** Data of AD-PSO coexistence cases in literature.

Author	Shivam Kapila, et al. (76)	TA Kouwenhoven, et al. (77)	A Docampo, et al. (78)	Beer WE, et al. (74)	Bozek A, et al. (79)	Kelly Barry, et al. (75)
Disease name	PD	PD	PD	Concomitant atopic dermatitis and psoriasis	Concomitant atopic dermatitis and psoriasis	Concomitant atopic dermatitis and psoriasis
Study type	Single-center retrospective study	a single-center cohort study	multicentre prospective study involving 12 hospitals	Single-center prospective study	prospective, observational, two-center study	Single-center retrospective study
Country	Australia	the Netherlands	eastern Spain	UK	Poland	USA
Diagnostic criteria for AD	UK	Hanifin and Rajka criteria	/	/	UK	/
n.	44	15	14	45	38	30
Male/Female ratio	19:25	2:13	10:4	20:25	25:13	47%:53%
Age (year)	/	7.4	7	29.9 (1-77)	6.5	47.4 (11-84)
Age at onset (year)	1.18	4.9	5	/	/	/

### Eczematous psoriasis

Eczematous psoriasis was first categorized into primary and secondary forms by Ebstein et al. in 1991 (88). Primary eczematous psoriasis is described as an endogenous condition that frequently affects areas such as the groin and axilla, whereas secondary eczematous psoriasis is associated with external factors like irritants and allergens (88). The diagnosis of eczematous psoriasis requires the presence of characteristic psoriatic stigmata. A recent study involving 20 patients clinically diagnosed with psoriasis revealed that 15% were initially misdiagnosed with eczematous dermatitis rather than psoriasis, while 35% exhibited a psoriasis phenotype with eczematous features, a condition termed “eczematized psoriasis” (89). Clinical observations indicate that patients with eczematized psoriasis tend to be older than those with classical plaque psoriasis and display a mixed phenotype with overlapping features of both psoriasis and eczema (90).

### Eczema in psoriatico

Kolesnik M. reported that “eczema in psoriatico (EIP)” was a psoriasiform dermatitis characterized by neutrophils and plasma exudation in parakeratosis, lymphocytic exocytosis in the epidermis, full-thickness spongiosis, or formations of spongiotic vesicles, with overlapping features of both psoriasis and contact dermatitis (91). Although triggers for eczema or psoriasis may vary based on life stages, skin regions, and factors such as medication and trauma, a study revealed distinct differences between these conditions in terms of the intensity and pattern of parakeratosis, type of acanthosis, absence of granular layer loss and thinning of the suprapapillary plate, configuration of rete ridges, concentration of lymphocytic infiltration in the papillary dermis, and presence of papillary dermal edema, distinct from the occurrences of lymphocytic exocytosis, spongiosis, and spongiotic vesicles. These differences were observed not only in the palmoplantar area but also in other regions such as the rest of the palms and soles and flexural areas. Consequently, the authors contend that “eczema in psoriatico” is a separate entity from psoriasis (92).

### Psoriasiform paradoxical reactions and eczematous paradoxical reactions

Treatment of eczema with dupilumab can lead to the development of psoriasiform eruptions, known as psoriasiform paradoxical reactions (P-PRs). Conversely, cases of eczematous paradoxical reactions (E-PRs) have been described in patients with psoriasis treated with biologics. Factors common to some reported cases included a prior history of atopy or psoriasis, eosinophilia, and raised serum IgE. A personal history of atopy may help predict patients who may experience E-PRs, with 48.98% (24/49) of E-PR cases occurring in patients with a personal history of atopy (dermatitis, allergies, or asthma) (93–95). In biologic-treated patients with psoriasis, the risk of paradoxical eczema was lowest in those receiving IL-23 inhibitors. Increasing age, female sex, and a history of atopic dermatitis or hay fever were associated with a higher risk of paradoxical eczema, although the overall incidence was low (96).

### Eczema-psoriasis overlap/psoriasis eczema overlap/PsEma/PD

In 2001, a retrospective analysis of 1262 pediatric PSO patients found 55 cases (4.3%) displaying Eczema-psoriasis overlap (97). Another study of 276 pediatric PSO cases identified 14 cases (5.1%) of Psoriasis eczema overlap (98), However, these studies merely noted the overlap without naming this overlap. In 2005, the term “PsEma” was introduced for the first time by researchers who conducted a prospective study on 100 PSO patients, discovering that 20% of the cohort could be diagnosed with an “intermediate state” exhibiting trait from both PSO and eczema histories. The team believed that “PsEma” is an overlapping state showing PSO and eczema features in clinical, histological, molecular, biological, and treatment responses (99). In 2012, the overlapping condition manifesting clinical characteristics of both PSO and AD was termed Psoriasis-Dermatitis (PD). This claim was supported by a study that involved 170 patients under 12 years old with papulosquamous lesions, identifying 44 PD cases (25.9%) (84). In 2019, one single-center clinical research involving 410 pediatric PSO patients noted 15 PD cases (3.7%) (85), and subsequent researchers reported 14 PD cases (21.5%) out of 65 PSO patients (86). The reported prevalence of PD varies among studies, possibly due to the absence of a unified diagnostic criterion for PD, results from different countries. Recent studies have attempted to define this emerging clinical entity in pediatric populations, but there is still limited information regarding PD in adults. Since this condition is still being characterized, there is a notable absence of conclusive data on its clinical and epidemiological features, as well as the varying responses to different treatments (100–103).

This recent study aimed to clarify the clinical manifestations of PD in adult patients. Researchers followed 26 PD patients over a 12-month period, comparing their clinical profiles to control groups of PSO and AD patients. The results revealed that PD patients tended to experience disease onset at a younger age and had a shorter symptom duration compared to those with PSO and AD. Furthermore, areas such as the scalp, feet, and genitals were more frequently affected in PD than in AD. The study suggests that PD may lie on a continuum between PSO and AD, rather than representing a distinct disease entity (104). Another study explored the clinical progression of PD in children, a condition presenting overlapping features of AD and PSO. In a cohort of 24 pediatric patients diagnosed with PD, after a median follow-up of 31 months, 83.3% evolved into a definitive form of either PSO or AD (38.9% developed AD and 44.4% PSO). Younger age and a family history of PSO were predictors for progression to AD. The findings suggest that most pediatric PD cases will eventually develop into AD or PSO, necessitating long-term clinical follow-up for accurate diagnosis. This finding suggests that PD may be an early form of AD or PSO rather than a separate entity (105).

The nomenclature regarding the coexistence or overlap of AD and PSO in the literature is notably disorganized and often conflated. However, for the sake of academic rigor, it is essential to distinguish and standardize our terminology. Coexistence should be defined as the simultaneous presence of AD and PSO in one patient, encompassing two scenarios: both conditions occurring independently at different times or the concurrent presentation of typical AD and typical PSO lesions in the same individual. Overlap refers to the concurrent manifestation of specific clinical features from both AD and PSO, making a definitive distinction challenging; in such cases, a clear diagnosis of either typical AD or typical PSO cannot be established. For cases of overlap, it is proposed that this condition be aptly termed “Psoriatic Dermatitis” (PD). This terminology succinctly encapsulates the overlapping features with both specificity and clarity.

### Complex immunopathology and potential mechanisms of coexistence between atopic dermatitis and psoriasis

Despite their distinct signaling pathways, significant overlap in gene dysregulation exists between AD and PSO, with studies indicating that 81% of gene disruptions in AD also occur in PSO (19). This suggests a shared genetic basis that may underpin the coexistence of these conditions. Additionally, genome-wide association studies (GWASs) have identified several overlapping risk loci for both diseases, although these loci can exhibit both concordant and discordant effects on disease susceptibility (106, 107). Although some have the same direction of effect, others (such as *rs28383201* mapping to *HLA-DRB1*, *rs55879323* within *FLG-AS1*, and *rs131952222* in intronic IL13) show opposite directions of effect, meaning that variants associated with an increased risk of PSO are associated with a lower risk of AD and vice versa (108). However, the co-occurrence of both diseases in the same individual is less frequent than expected based on their prevalence in the population (109–111), indicating that in addition to a genetic predisposition, the coexistence of the two conditions may be attributed to other contributing factors. Moreover, the paradoxical eczema systemic inflammatory proteome trends toward AD at a gene-set level and is detectable before the onset of the phenotype. This signature could be genetically determined (111). Using proteomic analysis, researchers identified that paradoxical eczema shares a systemic inflammatory signature with AD. Key findings revealed significantly reduced STAMBP protein expression in pre-biologic cases, coupled with the enrichment of eleven gene sets in post-biologic cases and ten gene sets in post-paradoxical eczema cases. These enriched pathways predominantly included cytokine and chemokine signaling networks, notably the KEGG cytokine-cytokine receptor interaction and KEGG chemokine signaling pathways, indicating substantial alterations in inflammatory signaling mechanisms during disease progression. Additionally, a polygenic risk score, based on 38 single nucleotide polymorphisms associated with these gene sets, suggests a genetic predisposition to paradoxical eczema (111). In fact, systemic therapies may be a trigger to promote the processes by which AD patients develop PSO or PSO patients develop AD. These processes can also happen regardless of whether a patient is undergoing systemic or topical therapies (112).

Research by Eyerich S et al. (113). has further elucidated the complex immunological interactions in patients with coexistent AD and PSO. They found that different T-cell subgroups, specifically Th1 and Th17 in PSO and Th2 in AD, can coexist within the same tissue, indicating a sophisticated level of immune regulation that might explain the clinical manifestations seen in these patients. This was observed particularly after epidermal challenges, where patients exhibited responses typical of AD rather than the expected psoriatic reactions, a phenomenon likely driven by antigen-specific T-cell subgroups. Moreover, IL-36 and β-defensin 2 (BD-2), which are markers of IL-17 signaling, have been found to be significantly elevated in PSO compared to AD, suggesting a pronounced role for Th17 cells in PSO pathogenesis (114). A recent study included 38 patients with both AD and PSO, as well as 41 patients with AD and 28 patients with PSO. The levels of TNF-α, IFN-γ, IL-2, IL-4, IL-5, IL-6, IL-8, IL-12, IL-17, IL-18, IL-22, IL-33, and TARC/CCL17 were measured. The study found that IL-17 levels were significantly higher in patients with both AD and PSO compared to those with AD or PSO alone, underscoring the potential role of Th17 cells in the overlap of these conditions (87). IL-17C is not specifically related to Th2 or Th17 immunity but amplifies keratinocyte-derived pro-inflammatory signals in murine and human models of both psoriasis and AE. Hence, IL-17C is a potential target to treat a broad variety of inflammatory skin diseases (ISDs) (115). However, the precise mechanism underlying the concomitant occurrence of PSO and AD remains elusive. The main immunopathogenesis of overlap AD and PSO is shown in **Figure 1**.

### Clinical and pathological features of concomitant PSO and AD

**Figure 3:**
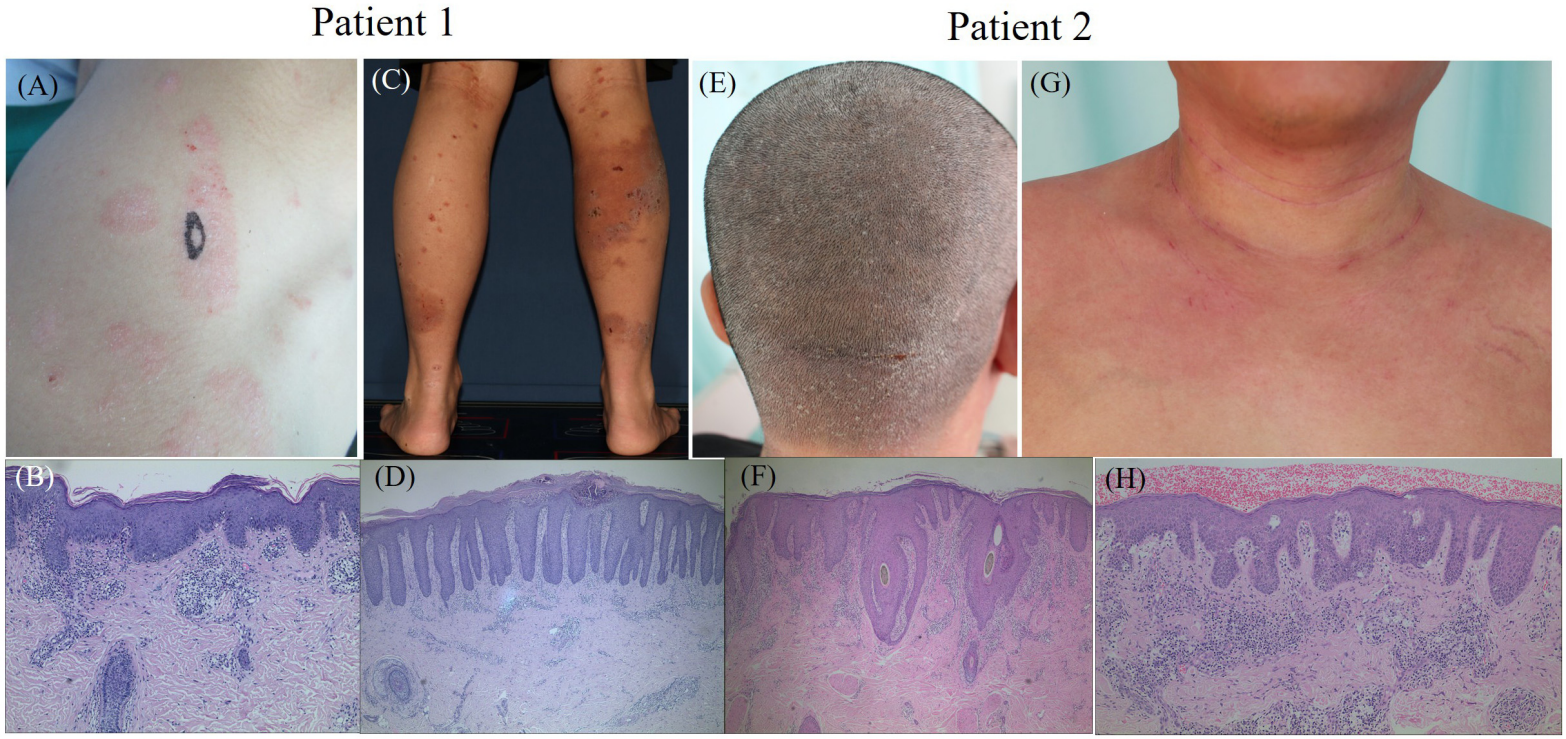
The clinical manifestations and histopathological results of two typical patients with PD. Patient 1. **(A)** plaque and scales on the right shoulder, **(B)** histopathology of the right shoulder (Black oval mark) showing parakeratosis, acanthosis with irregular elongation and infiltration of lymphocytes around small vessels in the superficial dermis, **(C)** erythema, erosion and crust on the right lower limbs, **(D)** histopathology of the right calf (Black oval mark) showing parakeratosis, acanthosis with regular elongation, kogoj microabscesses, infiltration of lymphocytes and eosinophils around small vessels in the dermis. Patient 2. **(E)** erythema and scales on the scalp, **(F)** histopathology of the scalp behind the pillow (Black oval mark) showing parakeratosis, acanthosis with irregular elongation, infiltration of lymphocytes, neutrophils and eosinophils around small vessels in the dermis, **(G)** erythema in the neck and chest, **(H)** histopathology of the chest (Black oval mark) showing parakeratosis, acanthosis with irregular elongation and infiltration of lymphocytes around small vessels in the dermis.

The prevalence of concomitant PSO and AD varies across studies, with some reporting a higher prevalence of concomitant PSO and AD in males (71.2% and 65.8%, respectively) (86, 87). However, female dominance was reported in other studies [55.6% (82), 56.8% (84), 86.7% (85), and 53% (83)]. Interestingly, coexistence is more frequently observed in boys and overweight individuals (87). These variations suggest that PSO and AD can manifest together in three primary scenarios: (1) simultaneous eruption of both diseases, which is rare; (2) alternate flaring, where the diseases erupt sequentially rather than concurrently, occurring occasionally; and (3) sequential development across different life stages, with AD typically presenting in childhood and subsiding for years before PS emerges in adulthood. This latter scenario is considered the most common (87). Histopathologically, eczematized psoriasis, which is characterized by features such as acanthosis, hypogranulosis, thinning of the suprapapillary epidermis, hyperparakeratosis, Munro’s microabscesses, alongside additional spongiosis, eosinophils, and serum crusts, is indicative of PD (90). Clinical manifestations with PD are shown in **Figure 3**.

Further histopathological evaluation provides deeper insights, especially in cases with clinical features of both AD and PSO. Typically absent in PSO (116), eosinophilic leukocytes are commonly found in AD. However, studies have reported the presence of eosinophils in 46% (117) and 18% (118) of psoriasis biopsy specimens. In our studies, dermal eosinophil infiltration was observed in 38.1% of PD patients (119). Additionally, features such as psoriasiform hyperplasia, parakeratosis, hypogranulosis, Munro’s microabscesses, and notably distorted capillaries in the papillary dermis are more prevalent in palmoplantar lesions of PSO than in those of eczematous dermatitis, though these differences did not reach statistical significance. Conversely, a significant proportion of PSO patients (76.5%) exhibited spongiotic vesicles (120). These observations support the concept that PD represents an intermediate histological state between PSO and AD (119).

### Molecular diagnostic advances in differentiating atopic dermatitis and psoriasis

Advances in molecular diagnostics have introduced refined methods to distinguish between AD and PSO, particularly in clinically ambiguous cases. A significant development by Eyerich et al. involved a molecular classifier (MC) using the biomarkers NOS2 and CCL27, which has proven effective in differentiating PSO from AD with high sensitivity and specificity (23, 121, 122). This classifier demonstrates notable diagnostic accuracy, even in cases where clinical presentation overlaps, and has shown high diagnostic efficacy in formalin-fixed, paraffin-embedded tissue samples, microbiopsies, and tape strips (123). Despite these promising results, the broader clinical adoption of this molecular tool remains limited due to its relatively recent introduction and requirement for specialized laboratory protocols.

Further molecular studies have examined gene expression profiles in patients presenting with overlapping features of AD and PSO, suggesting that PSO-associated genes, particularly NOS2, IL36G, and CCL27, predominate in ambiguous cases (124). In addition, a unique histological marker, known as rouleaux formation, has been observed with greater frequency in PSO compared to other dermatitides. This microscopic phenomenon, involving red blood cell aggregation into coin-stack formations due to plasma protein interactions, has a reported sensitivity of 68% and specificity of 75% for distinguishing PSO from other inflammatory skin conditions. However, given its presence in benign keratosis as well, rouleaux formation is considered a supplementary rather than definitive diagnostic feature (125).

The MC developed by Eyerich et al. (122), which utilizes NOS2 and CCL27, has shown diagnostic sensitivity and specificity exceeding 95% in a study cohort comprising both classic and atypical variants of PSO and AD. Subsequent research has reinforced the diagnostic utility of this classifier for differentiating PSO and AD based on molecular biomarkers, with a sensitivity of 92%, specificity of 100%, and an area under the curve (AUC) of 0.97 (123). These findings underscore the potential of molecular diagnostics to support differential diagnosis in clinical and outpatient settings, particularly for chronic inflammatory skin diseases where clinical presentation alone may be insufficient.

### Treatment of concomitant AD and PSO

The optimal treatment for patients with concomitant AD and PSO remains an active area of investigation. Recent phase II clinical trials have demonstrated the effectiveness and tolerability of the aryl hydrocarbon receptor (AHR) agonist tapinarof in both conditions, offering a promising topical option (126, 127). IL-23A has also been identified as a potential therapeutic target for patients with overlapping AD and PSO symptoms, highlighting its role in shared pathogenesis (128). Another approach involves MOR106, an antibody targeting IL-17C, which may be beneficial by modulating inflammatory pathways driven by both Th2 and Th17/Th22 cells (129). However, the efficacy and safety of these therapies still require validation through larger, more diverse trials.

Topical treatments alone have often been inadequate for concurrent AD and PSO, necessitating more robust systemic interventions. Common systemic therapies, such as immunosuppressants (methotrexate, azathioprine, cyclosporine) and phototherapy, are applicable to both conditions. More recently, JAK inhibitors and PDE4 inhibitors have shown promise due to their safety and efficacy profiles (1). Although biologics targeting specific T-cell subsets have shown limited success for coexisting AD and PSO, broader T-cell suppression strategies—especially those targeting the IL-23/Th17 axis—may offer a more effective approach (124). Case reports on upadacitinib suggest its effectiveness in patients with dual conditions (130), with similar results observed in a recent study (119). Despite these findings, larger studies are required to confirm the viability of JAK inhibitors in this context.

Studies indicate that biologics targeting IL-17 or IL-23 may be more effective than traditional treatments, such as methotrexate and cyclosporine (104). Some clinicians have advocated for dual biologic therapy to manage concurrent AD and PSO, with reports of significant therapeutic benefits (131–133). However, the financial implications and long-term safety of combining biologics warrant careful consideration and further study.

## Conclusion and perspectives

AD and PSO are two distinct diseases in terms of mechanisms, but they can coexist in clinical settings, and some subtypes deserve attention, which may be pedigree diseases. However, their coexistence within the same clinical profiles presents a complex challenge, highlighting the nuanced interplay of immune responses in dermatological conditions. We propose naming the condition characterized by their overlap as psoriasis dermatitis (PD). Numerous questions remain to be addressed, such as the appropriate assessment methods – should both PASI and EASI be employed concurrently, or is there a possibility to devise a novel evaluation technique, perhaps PEASI? What are the precipitating factors contributing to the coexistence of these conditions, and is there a need for prospective studies to uncover them? What are the underlying immunological mechanisms when these diseases coexist, and which therapeutic strategy is optimal? Should we administer two separate biologic agents simultaneously, or utilize a single medication capable of treating both conditions, such as JAK inhibitors or PDE4 inhibitors? Ongoing and future research is essential to unravel the complex pathogenesis of PD, refine diagnostic criteria, and optimize therapeutic approaches. Such efforts will not only enhance our understanding but also improve the quality of life for patients suffering from this challenging dermatological confluence.
